# Determinants of respiratory diseases in East Sikkim

**DOI:** 10.1186/1756-0500-6-356

**Published:** 2013-09-08

**Authors:** Ankur Barua, VK Tiwari, Santosh P Kesari

**Affiliations:** 1 Department of Community Medicine, Sikkim-Manipal Institute of Medical Sciences (SMIMS), Sikkim, India; 2 Department of Chest Medicine and Tuberculosis, Sikkim-Manipal Institute of Medical Sciences (SMIMS), Sikkim, India; 3 Department of Community Medicine, International Medical University (IMU), No. 126, Jalan Jalil Perkasa 19, Bukit Jalil 57000, Kuala Lampur, Malaysia

**Keywords:** Case, Control, Risk, Factors, Socio-demographic, Correlates

## Abstract

**Background:**

Due to the difficult geographic terrain with lack of roads and transport, the Sikkim State in India finds difficulties in contending the respiratory diseases especially during the rainy seasons.

**Findings:**

A case–control study was conducted for two months at the Central Referral Hospital of East Sikkim involving 110 individuals in the age group of 10 years and above. Due to feasibility constraints, 55 cases and 55 controls were selected by applying the non-probability sampling method with age and sex matching. The collected data were tabulated and analyzed by using the SPSS (Statistical Package for Social Sciences) version 10.0 for windows. Findings were expressed in terms of proportion, Chi Square Test and Multiple Logistic Regression Analysis. Here, *p*-value <0.05 was considered as statistically significant. This study revealed that the presence of overcrowding, chronic exposure to allergens, smoking habits, chronic respiratory illnesses within last 5 years, family history of chronic respiratory illnesses and mental illnesses were independently associated with respiratory diseases.

**Conclusion:**

This study should be replicated in other parts of Sikkim to obtain more confirmatory evidence on determinants of respiratory diseases.

## Findings

### Research hypothesis

The change in epidemiology and clinical spectrum of respiratory illnesses is now a global concern. Respiratory diseases are often found associated with infections, infestations, air pollution, tobacco smoking and environmental tobacco exposure [1-3]. The indoor pollutants such as paints, house hold cleaning devices, allergens, house dust, mites, fungi, dampness, humidity, congestion and combustion fumes from cooking fuel (wood, coal, kerosene) are likely to cause respiratory illnesses [4,5]. With this background, a study was conducted to identify the determinants of respiratory diseases in East Sikkim.

## Methods

A case–control study was conducted for two months (from 1^st^ September to 31^st^ October 2007) to identify the determinants of respiratory diseases in East Sikkim. Individuals who reported with respiratory diseases at Central Referral Hospital were selected as cases and their controls were selected from corresponding villages of East Sikkim. The total number of patients visited this hospital during these two months was 325.

In this study, the individuals who were diagnosed by a registered medical practitioner to be suffering from any respiratory illness (either acute or chronic) were considered as cases. Individuals, who were not suffering from any diagnosed respiratory illnesses, were considered as controls. The age and sex matching was done to pair up each control with its corresponding case. The sample size was calculated manually and verified by the statistical package of Epi-Info version 5.0. The parameters considered for the sample size calculation are as follows: The Confidence Interval was set at 95%, power at 80% and smoking habits was considered as the major exposure. Here, the prevalence of exposure (smoking habits) in controls was taken as 10% while that of cases was 35% and Odds Ratio closest to 1 was estimated to be 4.85. A provision of extra 10% of sample size in both the groups was kept to compensate for the non-respondents. Hence, the final sample size included 55 cases and 55 controls. So, a total of 110 individuals in the age group of 10 years and above, who were permanent residents of East Sikkim formed the study sample.

This was a community oriented project tailored for undergraduate medical curriculum. Hence, it had a significant amount of feasibility constraints and time restrictions. Due to these constraints the researchers had to work with a smaller sample size by compromising the power and expected Odds Ratio. This small sample size restricted the subgroup analysis of adolescent and adult age groups and also a further subgroup analysis of acute and chronic respiratory diseases.

This project was approved by both the research and ethical committees of the Indian Council of Medical Research (ICMR) and the Sikkim-Manipal Institute of Medical Sciences (SMIMS). Since, this is not an intervention study, both these committees of the respective institutions approved the administration of informed verbal consent during data collection. The other intention was to go green and save paper as well. Hence, in this study, none of the participants were subjected to any kind of intervention by the investigators. The data collected was kept anonymous and confidentiality was maintained throughout the study including the final research report. In a situation when participants were under the age of 18 years, the investigators obtained consent for their participation in this study from the respective parents or guardians.

All individuals in the age group of 10 years and above who gave informed verbal consent to participate in this study were included. Children below 10 years were excluded from this study. The individuals, who were non-cooperative and did not give informed verbal consent to participate in the study, were also excluded. There were altogether 10 individuals who did not give informed verbal consent to participate in this study. Since, prior provision was made in the sample size calculation for these non-respondents; we expect a minimal impact of this on the results.

The selection of the cases was done at the Department of Chest Medicine & Tuberculosis of the Central Referral Hospital while the investigators surveyed the corresponding community for controls. The controls were selected from the same village as cases. The individuals who visited central referral hospital with the respiratory illnesses were interviewed and examined thoroughly and the findings were recorded on a pre-tested proforma which was designed for the study. All the individuals were subjected for routine x-ray, blood examination and sputum examination. All the individuals suffering from respiratory illnesses (excluding tuberculosis) were subjected to PFT (Pulmonary Function Test) by an Automated Computerized PFT Machine. The most important parameters of spirometry used in this study was the forced vital capacity (FVC), which was the volume delivered during an expiration made as forcefully and completely as possible starting from full inspiration. Another important parameter was the forced expiratory volume (FEV) in one second, which was the volume of air delivered in the first second of an FVC maneuver. There are minor differences between the two most recent American Thoracic Society (ATS) and European Respiratory Society (ERS) statements, except that the ERS statement includes absolute lung volumes while the ATS does not. In this study, the updated criteria by the American Thoracic Society (ATS) were followed for all diagnostic purposes.

Paracentesis was done in cases of pleural effusion and the aspirated fluid was examined for biochemical, cellular, cytological (>50 years), microbiological examination including AFB (Acid Fast Bacilli). Selected individuals from the corresponding communities of East Sikkim, who were not suffering from any respiratory illnesses, were interviewed and examined as controls. Thorough clinical examination, investigations and free referral services were provided to all the participants:

The collected data were tabulated and analyzed by using the SPSS (Statistical Package of Social Sciences) version 10.0 for windows. Findings were expressed in terms of proportion. Chi Square Test was applied for Univariate Analysis and Multiple Logistic Regression Analysis was done to study the independent effect of each variable over the outcome. In this study, *p*-value <0.05 was considered as statistically significant.

## Results

In this study population, age and sex distribution were used as matching criteria. Hence, the distribution of these two parameters was similar in both the case and control groups. Table 1 shows the socio-demographic profile of the study population. Majority of the participants 70(63.6%) were Hindus, 60(54.5%) were married, 90(81.8%) were literate, 37.3% had smoking habit and 45(40.9%) were using fossil fuels. Though majority of the study population used cooking gas (59.1%), but the commonly used fossil or smoke producing fuels by 45(40.9%) of study population included wood, coal and kerosene. Chronic respiratory illness within last 5 years was present among 38(34.5%) participants. Among the cases, 28(50.9%) were suffering from Chronic Obstructive Pulmonary Diseases in the form of emphysema or chronic bronchitis, 13(23.6%) had acute respiratory diseases like pneumonia or influenza, 11(20.0%) had acute episodes of Chronic Bronchial Asthma, while 7(12.7%) were diagnosed of active Pulmonary Tuberculosis. Some of the participants were suffering from more than one disease.

**Table 1:** Baseline information

***Socio-demographic profile***	***Category***	***Number***	***Percentage***
		***N = 110***	***(%)***
***Religious status***	***Hindu***	70	63.6
	*Buddhist*	30	27.3
	*Christian*	10	9.1
***Marital status***	***Married***	60	54.5
	*Unmarried/ Widowed/ Separated/ Divorced*	50	45.5
***Literacy status***	***Literate***	90	81.8
	*Illiterate*	20	18.2
***Use of fossil/ smoke producing fuel for cooking***	***Absent***	65	59.1
	***Present***	45	40.9
***H/o chronic respiratory illness within last 5 years***	***Absent***	72	65.5
	***Present***	38	34.5
***Family H/o chronic respiratory illness within last 5 years***	***Absent***	78	70.9
	***Present***	32	29.1
***Smoking habit***	***Absent***	69	62.7
	*Present*	41	37.3
***H/o passive smoking***	***Absent***	88	80.0
	*Present*	22	20.0
***History of psychiatric illness attributing to respiratory distress***	***Absent***	94	85.5
	*Present*	16	14.5

The Univariate Analysis in Table 2 revealed a significant association between the respiratory diseases and socio-demographic correlates such as marital status, type of fuel used, literacy, smoking habits, passive smoking or environmental tobacco smoke, overcrowding, history of chronic respiratory illnesses within last 5 years, family history of chronic respiratory illnesses within last 5 years, pets at home, history of chronic exposure to allergens in household, family history of chronic exposure to allergens, history of parasitic diseases and mental illnesses on the basis of statistical interpretations.

**Table 2:** Determinants of respiratory diseases: univariate analysis

***Risk factors of respiratory diseases***	***Category***	***OR***	***95% CI of OR***	***p-value***
		***(Unadjusted)***	***(Unadjusted)***	
***Marital status***	***Married***	1.00	-	*-*
	*Unmarried/ Widowed/ Separated/ Divorced*	0.48	0.17-0.79	0.031*
***Overcrowding***	***Absent***	1.00	-	*-*
	*Present*	14.2	9.2-17.6	0.0001*
***Literacy status***	***Literate***	1.00	-	*-*
	*Illiterate*	5.2	2.6-8.7	0.026*
***H/o chronic exposure to allergen***	***Absent***	1.00	-	*-*
	*Present*	8.2	4.3-11.8	0.003*
***Family H/o chronic exposure to allergen***	***Absent***	1.00	-	*-*
	*Present*	3.8	1.3-6.9	0.037*
***Use of fossil/ smoke producing fuel for cooking***	***Absent***	1.00	-	*-*
	***Present***	8.4	2.3-11.4	0.038*
***H/o chronic respiratory illness within last 5 years***	***Absent***	1.00	-	*-*
	***Present***	10.6	5.6-13.2	0.012*
***Family H/o chronic respiratory illness within last 5 years***	***Absent***	1.00	-	*-*
	***Present***	2.3	1.5-6.3	0.001*
***Smoking habit***	***Absent***	1.00	-	*-*
	*Present*	3.4	2.3-7.7	0.001*
***H/o passive smoking***	***Absent***	1.00	-	*-*
	*Present*	7.7	5.1-9.4	0.047*
***Pets at home***	***Absent***	1.00	-	*-*
	*Present*	2.4	1.4-5.6	0.046*
***H/o parasitic diseases affecting lung***	***Absent***	1.00	-	*-*
	*Present*	10.7	2.7-13.8	0.037*
***History of psychiatric illness attributing to respiratory distress***	***Absent***	1.00	-	*-*
	*Present*	8.2	4.6-12.3	0.0001*

* p value <0.05 is considered as significant.

In this study, the regular and ex-smokers were considered as smokers while the occasional and those who never smoked a single cigarette in life were considered as non-smokers. Here, regular smoker – consuming at least one pack for at least two times a week, ex-smoker – did not consume a cigarette in the last one year, occasional – consuming less than one pack for less than two times a week and non-smoker – never consumed cigarettes in life. These criteria were developed with reference to a study conducted by Hämäläinen J. et. al. during 2001 [6].

Chronic respiratory diseases are chronic diseases of the airways and other structures of the lung. Some of the most common chronic respiratory diseases are bronchial asthma, chronic obstructive pulmonary disease, occupational lung diseases and pulmonary hypertension. However, a comprehensive list of common chronic respiratory diseases is provided by the World Health Organization using the International Classification of Diseases (ICD-10) criteria [7,8].

The common parasitic diseases affecting the respiratory system in this population were hydatid cyst, chronic schistosomiasis, strongyloides stercoralis and the pulmonary phases of nematode infestations causing acute eosinophilic pneumonia. Cases of tropical pulmonary eosinophilia were associated with refractory bronchial asthma. Most of these parasitic diseases were initially diagnosed by detecting eggs or larvae in stool, sputum, pleural fluid or tissue and were later confirmed by serological investigations. Medical report issued by a registered medical practitioner was used as evidence for parasitic diseases.

The history for chronic exposure to allergen in household was obtained from the cases in the hospital through a detailed check-list of allergens and their continuous exposure in the household for at least 6 months. However, during the community survey this history was verified with direct inspection of the household conditions for the controls.

Pulmonary disease such as Bronchial Asthma is a psychosomatic disorder vulnerable to exacerbations precipitated by psychological factors. In this study, Anxiety Neurosis and Hysteria were found to be associated with acute exacerbation of Chronic Bronchial Asthma in four cases. Depression was associated with poorer adherence with asthma treatment leading to acute episodes in two cases. However, depression was also associated with six cases of Chronic Obstructive Pulmonary Diseases and two cases of pulmonary tuberculosis. Paradoxical Vocal Cord Dysfunction (PVCD), characterized by abnormal vocal cord adduction during inspiration, was also present in one patient with Chronic Bronchial Asthma.

The Multiple Logistic Regression Analysis in Table 3 revealed that the presence of overcrowding, history of chronic exposure to allergen in the household, history of chronic respiratory illness within last 5 years, family history of chronic respiratory illnesses, smoking habits and history of mental illnesses were independently associated with respiratory diseases in this community.

**Table 3:** Determinants of respiratory diseases: multiple logistic regression analysis

***Risk factors of respiratory diseases***	***Category***	***OR***	***95% CI of OR***	***p-value***
		***(Adjusted)***	***(Adjusted)***	
***Marital status***	***Married***	-	-	*-*
	*Unmarried/ Widowed/ Separated/ Divorced*	1.9	0.7-2.4	*0.559*
***Overcrowding***	***Absent***	-	-	*-*
	*Present*	1.7	1.2-3.6	0.0001*
***Literacy status***	***Literate***	-	-	*-*
	*Illiterate*	4.8	0.6-5.7	*0.245*
***H/o chronic exposure to allergen***	***Absent***	-	-	*-*
	*Present*	8.1	5.3-9.8	0.026*
***Family H/o chronic exposure to allergen***	***Absent***	-	-	*-*
	*Present*	0.2	0.1-1.9	*0.230*
***Use of fossil/ smoke producing fuel for cooking***	***Absent***	-	-	*-*
	***Present***	0.5	0.3-3.4	*0.550*
***H/o chronic respiratory illness within last 5 years***	***Absent***	-	-	*-*
	***Present***	3.8	1.6-7.2	0.002*
***Family H/o chronic respiratory illness within last 5 years***	***Absent***	-	-	*-*
	***Present***	5.3	1.4-8.3	0.011*
***Smoking habit***	***Absent***	-	-	*-*
	*Present*	8.7	3.5-9.7	0.042*
***H/o passive smoking***	***Absent***	-	-	*-*
	*Present*	0.3	0.1-3.4	*0.228*
***Pets at home***	***Absent***	-	-	*-*
	*Present*	0.2	0.1-2.6	*0.192*
***H/o parasitic diseases affecting lung***	***Absent***	-	-	*-*
	*Present*	0.3	0.1-13.8	*0.321*
***History of psychiatric illness attributing to respiratory distress***	***Absent***	-	-	*-*
	*Present*	8.0	3.4-11.3	0.0001*

* p value <0.05 is considered as significant.

## Discussions

A survey conducted among adults in South Africa by Wichmann J and Voyi KVV during 2005, revealed low prevalence rates for doctor diagnosed asthma (3.7%), chronic bronchitis (2.4%), emphysema/bronchitis (4.2%) and TB (2.7%). Among the clinically diagnosed cases, only 1.7% of the respondents reported using asthma medication while 0.5% was using TB medication [9]. In contrast to this, a significantly high prevalence of chronic respiratory illnesses during the last 5 years among the participants or their family members was observed in our study.

The multiple logistic regression analyses from the study by Wichmann J and Voyi KVV during 2005, revealed that age and race as the non-modifiable predictors. It was found that the prevalence of respiratory symptoms and diseases could be diminished in South Africa through health promotion predictors such as having a medical aid and improved health education. The modifiable risk factors identified in this study were households going hungry, years smoked, households with smokers, exposure at work to fumes, smoke and dust. These risk factors should be controlled in order to reduce the prevalence rates of respiratory symptoms and diseases in the community [9]. These determinants of respiratory illnesses were similar to our study findings.

A secondary data analysis during 2001 from the “Health and Social Survey Report of 1998” in Canada by Lévesque B et. al. [10] revealed that the prevalence of wheezing was 5.4% across all age groups. A total of 30,386 individuals participated in the study. Wheezing was associated with asthma, allergies, chronic bronchitis and emphysema. A low familial income and tobacco smoking were associated with wheezing, asthma, chronic bronchitis and emphysema. Passive smoking was associated with wheezing whereas the presence of carpets at home was associated with wheezing and asthma. This study emphasized the role of reducing tobacco smoking in the prevention of chronic respiratory diseases [10]. These determinants of respiratory illnesses were also similar to our study findings.

### Limitation

Due to feasibility and time constraints, the sample size was restricted to 110. Subgroup analysis to identify the determinants of individual respiratory illnesses could not be done due to the restriction of sample size. Hence, the factors found to be associated with respiratory illnesses in this study are general and non-specific to any particular respiratory illness.

## Conclusion

Due to restricted sample size, this study was unable to identify the determinants of any specific respiratory illness in the study population. This could pose a hindrance in the formulation of preventive strategies. In order to improve generalizability, this study should be replicated in other parts of Sikkim to obtain more confirmatory evidence on determinants of respiratory diseases. However, it is envisaged that the results of this study will draw attention to the socio-demographic and environmental risk factors and help in planning an integrated intervention program for the prevention of respiratory illnesses in the community.

### Consent statement

This project was approved by both the research and ethical committees of the Indian Council of Medical Research (ICMR) and the Sikkim-Manipal Institute of Medical Sciences (SMIMS). Since, this is not an intervention study both these committees of the respective institutions approved the administration of informed verbal consent during data collection. The other intention was to go green and save paper as well. Hence, in this study, none of the participants were subjected to any kind of intervention by the investigators. The data collected was kept anonymous and confidentiality was maintained throughout the study including the final research report. In a situation when participants were under the age of 18 years, the investigators obtained consent for their participation in this study from the respective parents or guardians.

## Competing interests

Financial competing interests.

This project was sponsored by the Indian Council of Medical Research (ICMR), New Delhi during February 2007 towards research development at MBBS level. A fund of Rs. 5000.00 (INR) and a certificate was awarded for this research project to the third year MBBS student Mr. Santosh P. Kesari. His research guides and technical advisors were Dr. Ankur Barua and Dr. V.K. Tiwari. The final project report was approved and accepted by the ICMR, New Delhi. The funding agency of ICMR, New Delhi had encouraged the researchers to go ahead with the publication of this project report and there is no conflict of interest of any kind.

• In the past five years have you received reimbursements, fees, funding, or salary from an organization that may in any way gain or lose financially from the publication of this manuscript, either now or in the future? Is such an organization financing this manuscript (including the article-processing charge)? - NO.

• Do you hold any stocks or shares in an organization that may in any way gain or lose financially from the publication of this manuscript, either now or in the future? - NO.

• Do you hold or are you currently applying for any patents relating to the content of the manuscript? Have you received reimbursements, fees, funding, or salary from an organization that holds or has applied for patents relating to the content of the manuscript? - NO.

• Do you have any other financial competing interests? - NIL.

Non-financial competing interests.

• Are there any non-financial competing interests (political, personal, religious, ideological, academic, intellectual, commercial or any other) to declare in relation to this manuscript? – Academic career growth.

## Authors’ contributions

AB conceived of the study, and participated in its design and coordination, performed the statistical analysis and helped to draft the manuscript. SPK carried out the data collection and drafted the manuscript. VKT participated in the design of the study and helped to draft the manuscript. All authors read and approved the final manuscript.
